# Metaconcrete: An Experimental Study on the Impact of the Core-Coating Inclusions on Mechanical Vibration

**DOI:** 10.3390/ma16051836

**Published:** 2023-02-23

**Authors:** Meisam Ansari, Christin Zacharias, Carsten Koenke

**Affiliations:** Institute of Structural Mechanics, Bauhaus-Universität Weimar, 99423 Weimar, Germany

**Keywords:** metaconcrete, damping aggregate, mesoscale model, vibration absorber, multi-TMD

## Abstract

Resonance vibration of structures is an unpleasant incident that can be conventionally avoided by using a Tuned Mass Damper (TMD). The scope of this paper contains the utilization of engineered inclusions in concrete as damping aggregates to suppress resonance vibration similar to a TMD. The inclusions are composed of a stainless-steel core with a spherical shape coated with silicone. This configuration has been the subject of several studies and it is best known as Metaconcrete. This paper presents the procedure of a free vibration test conducted with two small-scaled concrete beams. The beams exhibited a higher damping ratio after the core-coating element was secured to them. Subsequently, two meso-models of small-scaled beams were created: one representing conventional concrete and the other representing concrete with the core-coating inclusions. The frequency response curves of the models were obtained. The change in the response peak verified the ability of the inclusions to suppress the resonance vibration. This study concludes that the core-coating inclusions can be utilized in concrete as damping aggregates.

## 1. Introduction

Dynamic forces such as wind, earthquakes, traffic loads, etc. affect structures and their components throughout their lifespan. Mainly two parameters can characterize these forces: their frequency and magnitude. When the frequency of a dynamic force is close to the natural frequency of a structure, the amplitude of the structure’s vibration increases substantially. This is known as the resonance vibration. According to [1], several measures could be taken to avoid this unpleasant phenomenon. One solution is a Tuned Mass Damper (TMD), an auxiliary mass that vibrates out of phase and counteracts the vibration of the structure.

This study focuses on engineered inclusions in concrete as an alternative solution to resonance vibration. The inclusions are coated steel balls and they are configured to suppress the vibration similar to a TMD. They are randomly distributed in concrete and consequently form a multi-TMD setup, which is incorporated into the structure. Therefore, it is essential to review the classical TMD and the multi-TMD setup briefly. This helps to understand the mechanism of a damper as well as the engineered inclusions.

### 1.1. A Short Review of TMD

**Figure 1:**
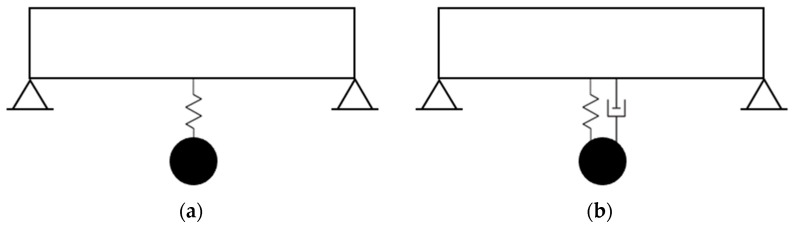
A single-span beam with a TMD at the mid-span. (**a**) Frahm’s vibration absorber (TMD without a self-damping). (**b**) TMD with a self-damping.

One of the earliest attempts to reduce the resonance vibration is the vibration absorber of Herman Frahm [2]. He mainly used his device on ships. He observed that a ship starts vibrating more as soon as its natural frequency is close to the frequency of its propelling machinery or its propeller. He described his invention as “an auxiliary body which is arranged within or on the main body whose vibrations are to be damped”. Figure 1a shows a schematic illustration of a single-span beam with an auxiliary body at the mid-span. The auxiliary body starts to vibrate by the vibration of the main body with a frequency as close as possible to the natural frequency of the main body. Because of the phase difference between the vibrations of both bodies, the impact of the vibration of the auxiliary body is in the opposite direction of the vibration of the main body. As a result of this, “the resonance of the main body is counteracted by the resonance of the auxiliary body”. His application for a patent for his invention was filed in 1909 by the United States Patent Office [2].

Frahm’s invention was what we know today as a damper without a self-damping component. However, a damper might also possess a self-damping component, which in turn could impact its efficiency in eliminating the vibration of the main body (Figure 1b). Den Hartog in [1] derived the formulation for such a configuration and described how one might find the “optimum damping ratio” of the damper. His optimization method was based on two ratios: the ratio of the damper’s mass to the main system’s mass (known as mass ratio), and the ratio of their frequencies. His study made a major contribution to the design of a TMD.

In Den Hartog’s classical optimization, the damping ratio of the main system was zero or close to zero [1]. However, real systems exhibit some degree of damping. [3] thoroughly reviewed the impact of the damping ratio of the main system on the optimization process. For a classical Single-Degree-Of-Freedom system (SDOF) with an attached absorber and a relatively small mass ratio, the damping of the main system showed only a small effect on the optimized parameter. [4] extended this result to any elastic body with an attached absorber that can be represented with the single mode under consideration without the absorber.

### 1.2. Multi-TMD Setup: A Solution to the Tuning Error

In real practical problems, it is hard to satisfy the optimized parameters of a TMD. [5] specified two main reasons for that:

(1) The frequency of the target mode of the main system is not always easy to be determined, particularly when the main system is a complex structure.
(2) The equivalent mass of the main system in the mode under consideration is hard to compute too.

The sensitivity of a TMD to the natural frequency of the main system, and to the damping ratio of itself, was argued in other works too [6,7]. Generally, the deviation from the optimized parameters, also known as the tuning error, is the main challenge in designing a TMD.

The authors of [7] highlighted that the damping ratio of a TMD may be intentionally made higher than the optimal value to reduce its sensitivity to the tuning error. Ref. [5] recommended increasing the optimized damping ratio by 20%.

**Figure 2:**
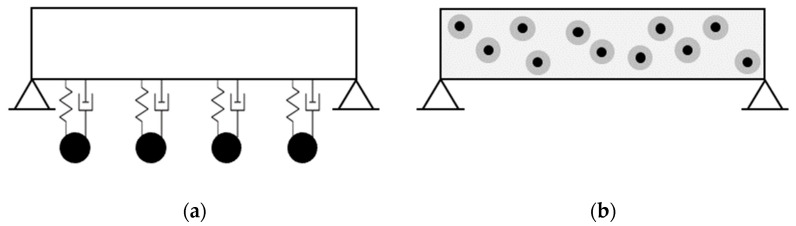
A single-span beam in a multi-TMD setup. (**a**) multiple TMDs distributed across the span. (**b**) distributed core-coating inclusions in concrete forming a multi-TMD setup.

Using multiple TMDs has been introduced as another solution for the sensitivity to the tuning error [6]. Figure 2a demonstrates a single-span beam with multiple TMDs. The masses of the individual TMDs are chosen to be equal. Their natural frequencies can be then tuned differently. In a classical setup, the natural frequencies of all TMDs are equal and tuned to a single natural frequency of the main system [6]. In another configuration, the natural frequencies of TMDs are distributed over a frequency range with the frequency of the main system being at the center [6,7,8,9]. When more than one natural frequencies of the main system are to be damped, TMDs can be grouped and tuned to each of them [10].

The study in [11] demonstrated the application of a multi-TMD setup to passive vibration control. It was shown that at a low damping ratio, multiple TMDs are significantly less sensitive to the tuning error. In addition, multiple TMDs were more effective than the single one in response to the wide-band excitation.

Several works have studied the dynamic characteristics and response of the multi-TMD setup. The complexity of these systems makes the interpretation of the result difficult. Ref. [12] provided a comprehensive study of the connected subsystems that led to a better understanding of their dynamic characteristics. A methodology based on general mathematical expression and matrix algebra was developed to solve the eigenvalue problem for the system with a simple configuration. The response of the system was then expressed in terms of the mobility and the impedances of the subsystems. The complementary work in [13] extended this approach to a more general and complex configuration. Both studies provided clear insight into the interaction between the main and the subsystems and why such a configuration can be effective in passive vibration control. 

The authors of [9] derived the frequency response function of a system with multiple TMDs. The dynamic effect of multiple TMDs on the response was remarkably described by an increase in the damping ratio of the main system, called “the equivalent damping”. Several properties of the equivalent damping were introduced, such as proportionality to the total mass of the TMDs, and the spacing between their natural frequencies. The equivalent damping was also proved to be independent of the damping ratio of the individual TMD.

The analytical study in [6] investigated the performance of a multi-TMD setup concerning three parameters: frequency range, damping ratio, and the number of TMDs. The common two-peak response curve of a system with a single TMD was considered. It was then demonstrated that the optimum tuning parameters for multiple TMDs could be found. The response curve of such an optimized multi-TMD setup would be flat between the common two peaks. The study could successfully correlate the design aspects of a multi-TMD setup with those of a single TMD.

Another contribution was made by [7]. A closed-form solution for the modal properties of the system with multiple TMDs was developed. The critical bandwidth was found, which is generally the smallest frequency range that a multi-TMD setup requires to make use of its multiplicity. A larger bandwidth can then result in higher robustness against the deviation from the tuning ratio. The study tabulated a procedure for the design of multiple TMDs.

### 1.3. Scope and Outline of the Work

The scope of this paper contains the utilization of the core-coating inclusions in concrete as damping aggregates. The goal is to investigate whether the inclusions can suppress mechanical vibration by functioning as dampers. In this regard, two main objectives are defined. Firstly, the damping ability of the core-coating inclusions is investigated by utilizing them individually as a TMD. Secondly, it is verified whether the inclusions can still function as dampers when they are randomly distributed in concrete.

Section 2 introduces the core-coating inclusions that are investigated in this research. Their mechanism and dynamic properties will be described.

In Section 3, the individual core-coating element will be regarded as a single TMD attached to a laboratory specimen of normal concrete. Their ability to suppress the vibration of the specimen is then investigated. This section mainly addresses the first objective highlighted earlier.

Section 4 is dedicated to the randomly distributed core-coating inclusions in concrete. The inclusions are considered to function as multiple TMDs tuned to a particular vibratory mode of the main system. Their impact on the vibration of the main system is investigated through numerical simulation. The second objective is addressed in this section.

In the final part, the conclusive findings are summarized.

## 2. The Core-Coating Inclusions

**Figure 3:**
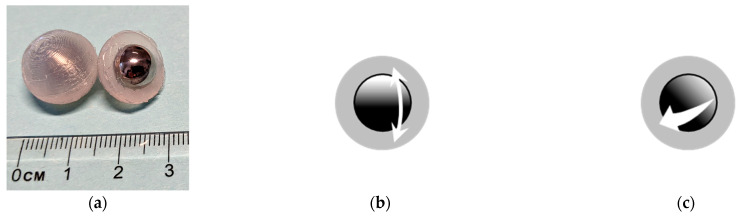
The core-coating inclusion and the oscillations of the core. (**a**) Core-Coating inclusion (**b**) translational oscillation of the core. (**c**) rotational oscillation of the core.

The engineered inclusions in this study are composed of a stainless-steel core with a spherical shape coated with silicone (Figure 3a). The utilization of these inclusions in concrete for a variety of purposes has been investigated by others too. For example, the author of [14] replaced the standard aggregate of regular concrete with silicone coated steel balls and introduced a new composite material named metaconcrete. The uniformly distributed inclusions were tuned such that their natural frequencies were close to the frequency of the applied harmonic wave. This resulted in the resonance of the inclusions and consequently the attenuation of the applied wave motion.

The study in [15] proposed the model of a metaconcrete thin plate composed of steel inclusions coated with rubber embedded in a concrete matrix. The thin plate showed great potential for passive vibration control in low frequencies.

The authors of [16] verified the attenuation property of metaconcrete through laboratory experiments with the cubic specimens of metaconcrete. The inclusions were composed of a spherical steel core coated with polydimethylsiloxane (PDMS). They were arranged inside the specimens in several symmetrical patterns.

The utilization of these inclusions is not limited to concrete. Similar engineered inclusions had also been used in the past under other names such as Locally Resonant Sonic Material (LRSM), Phononic Crystals (PnCs), etc. For example, [17] proposed a sound shielding structure composed of silicone-coated steel balls embedded in a resin slab. The configuration exhibited an increase in the acoustic transmission loss and formation of the band gaps. The numerical model of such a sonic barrier based on the mass-spring system was thoroughly developed and studied in [18].

The study in [19] presented the finite element model of a unit cell composed of a coated inclusion embedded in an epoxy matrix to study the resonance modes and the wave attenuation.

In the above works, the attenuation characteristics of the engineered inclusions in wave propagation, as well as sound shielding, were thoroughly studied, whereas the goal of this study is to investigate whether the inclusions can suppress the mechanical vibration at resonance by functioning as dampers. Another difference between the above works and this study is the distribution of the inclusions. In the above works, the inclusions were arranged uniformly. However, in this study, they are randomly distributed in concrete, similar to the normal aggregate.

### 2.1. Mechanism of the Inclusions

The inclusions’ mechanism relies very much on the high density of the core material and the elasticity of the soft coating. The coating is much softer and lighter than the core and it is wrapped around it. Hence, it forms a suspension enabling the core to oscillate when it is excited. The core can oscillate with translational and rotational motions [14,19] (Figure 3).

The oscillation of the core generates the restoring force required to suppress the vibration. The core has the largest displacement in the translational oscillation, which generates the greatest restoring force. This means that the most effective vibratory mode for the inclusion to work as a damper is the pure translational oscillation of the core [14]. A modal analysis is necessary to verify this critical mode and its frequency. The frequency of the translational mode is required for tuning the inclusions.

**Figure 4:**
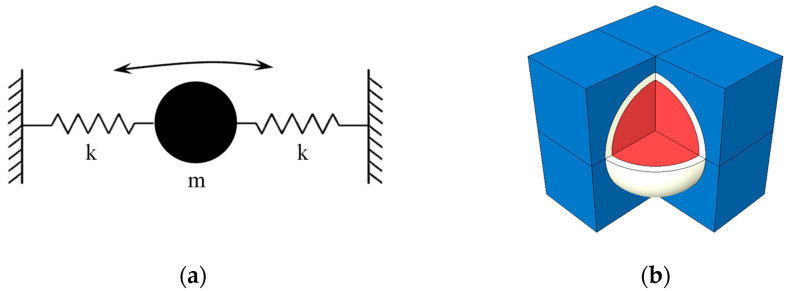
The core-coating inclusion and its equivalent models. (**a**) SDOF mass-spring system. (**b**) 3D FE-Model.

The simplest model for a modal analysis was proposed in [14]. The model idealizes the core-coating inclusion as a SDOF and represents it with a mass-spring composition (Figure 4a). The eigenfrequency in Hz is computed with

(1) $$f=\frac{1}{2\pi }\sqrt{\frac{3}{2}\;\frac{E_{c}}{R_{l}\;t\;\rho_{l}}}$$

where $E_{c}$ is the elasticity module of the coating material, t is the coating thickness, $R_{l}$ is the radius of the core and $\rho_{l}$ is the density of the core material. This model approximates the frequency of the translational mode and disregards the other vibratory modes [14].

The more accurate representation is a 3D FE-Model of the core-coating inclusion embedded in a cubic specimen of concrete, which was proposed in [14,19,20] (Figure 4b). The model exhibits a relatively large number of modes. By restraining the translational DOFs of the outer surfaces of the cube in the cartesian directions, the number of modes can be reduced. This makes it easier to identify the mode of interest, which is the translational vibration of the core along either of the Cartesian axis.

The modal analysis can also be conducted experimentally. For example, [17] measured the eigenfrequency of the core-coating inclusions by a laboratory dynamic test. A cubic specimen containing a single core-coating inclusion was suspended by rubber bands. The cube was excited in a particular frequency range by a small shaker. The applied harmonic force and the acceleration of the cube were measured by a force transducer and an accelerometer respectively. The frequency response of the cube in terms of the dynamic mass (ratio of force over acceleration) was plotted. The peak of the response graph corresponded to the eigenfrequency of the single inclusion in the cube.

The authors of [16] conducted an extensive study on the attenuative properties of metaconcrete. The eigenfrequencies of the inclusions were identified too. The inclusions were arranged in the cubic specimens in a regular lattice-like pattern. Each specimen contained a different number of inclusions. A signal with a linear frequency sweep was generated by a vibration speaker mounted on one side of the specimen. The time history of the traveled signal was recorded with the accelerometer at the opposite face. The Fourier transformation of the signal was then obtained to evaluate the eigenfrequencies of the inclusions.

### 2.2. Estimation of the Inclusions’ Natural Frequency

**Figure 5:**
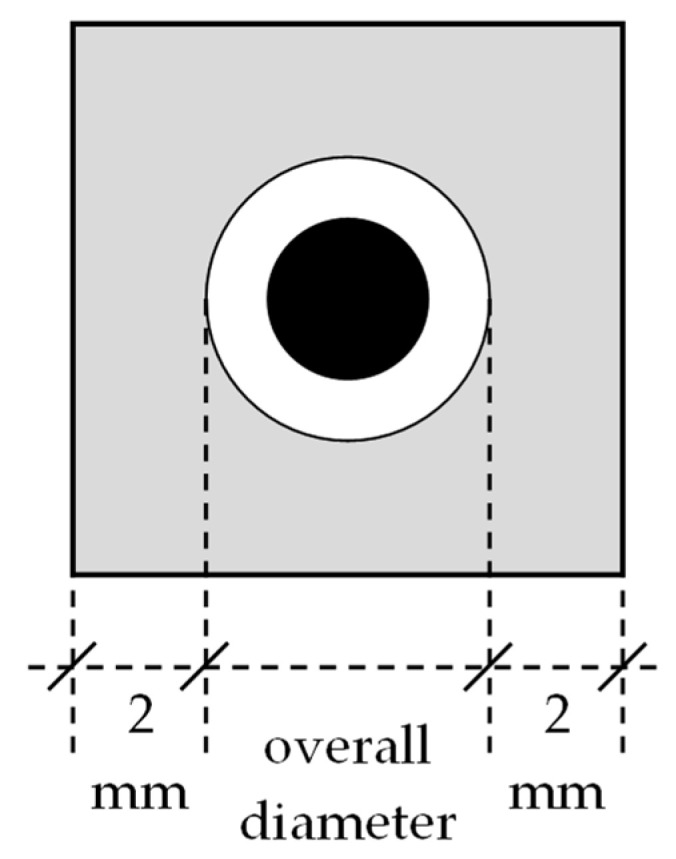
Dimensions of the FE-Model.

In this study, the translational eigenfrequency of the core-coating inclusion was estimated with the 3D FE-Model (Figure 4b), and its verification through a laboratory experiment is within the scope of future work. The 3D FE-Model of the cube with one core-coating inclusion at its center was created in Abaqus. Table 1 summarizes the mechanical properties of the materials used in the analysis. Table 2 provides the core-coating sizes used in this study. Figure 5 shows the dimensions of the FE-Model.

**Figure 6:**
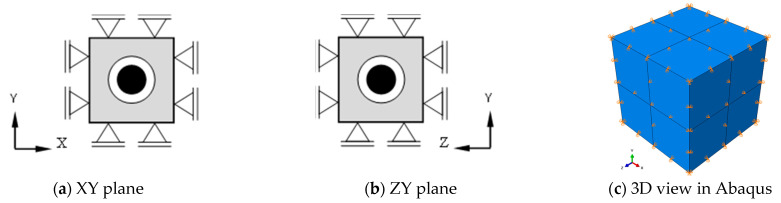
Boundary conditions of the 3D FE-Model (**a**,**b**): illustrative presentation, (**c**) Model in Abaqus.

The boundary conditions of the model are shown in Figure 6. As recommended in [14,19,20], the translational DOFs in XY and ZY planes were restrained to reduce the number of DOFs. This did not influence the translational motion of the core and its frequency.

The quadratic tetrahedral element of type C3D10 was used in the FE-Model. The global seed size was 1 mm and the “free” mesh technique in Abaqus was chosen to discretize the model.

“Natural frequency extraction” with “Lanczos” eigensolver in Abaqus was used to find the first 15 eigenvalues of the model.

The target mode shape was the translational motion of the core, which was found after the analysis by reviewing the extracted mode shapes. Table 2 provides the frequency of the target mode for the core-coating sizes.

## 3. Functioning as a Single TMD

The first objective of this study was to investigate the ability of the core-coating inclusions to function as a single TMD. It means whether we can tune them to a structural element to suppress its vibration. To achieve this objective, a “free vibration test” was conducted. The goal of the test was to obtain the damping ratio of the tested specimen. The damping ratio describes the decay of the free vibration. Therefore, it can easily describe how far the vibration is suppressed. This method was also employed in other studies, for example in [21]. In this regard, the specimen was tested two times: firstly, without the core-coating inclusions, and lastly with the inclusions attached to it. An increase in the damping ratio is evidence of the inclusions’ ability to suppress the vibration.

### 3.1. Description of the Test Setup

The experimental specimens were small-scale beams with the given specifications in Table 3. The beams were made out of concrete C35/45. The volume fraction of the components in the mix design is provided in Table 7. Cement type I was used in the mixture. The mass of the specimens was determined by simply weighing them with a weight scale.

**Figure 7:**
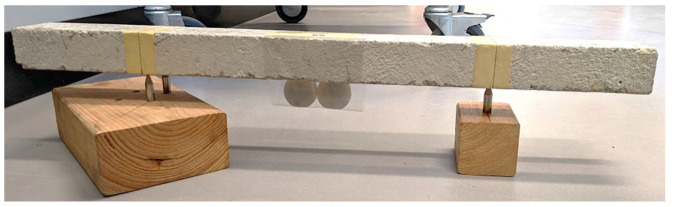
Point-Supporting the Beam in the Lab.

**Figure 8:**
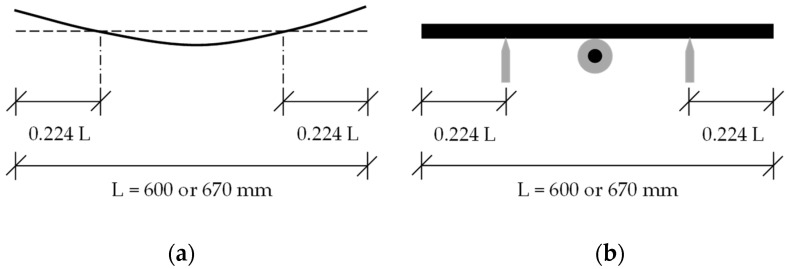
Alignment of the supports, (**a**) the 1st mode shape of beam with free ends; (**b**) point-supported beam with a core-coating element at mid-span.

The beams were point-supported as shown in Figure 7 and Figure 8b. The alignment of the supports was based on the first mode shape of the beam with free ends (Figure 8a). This arrangement minimized the impact of the supports on the vibration.

**Figure 9:**
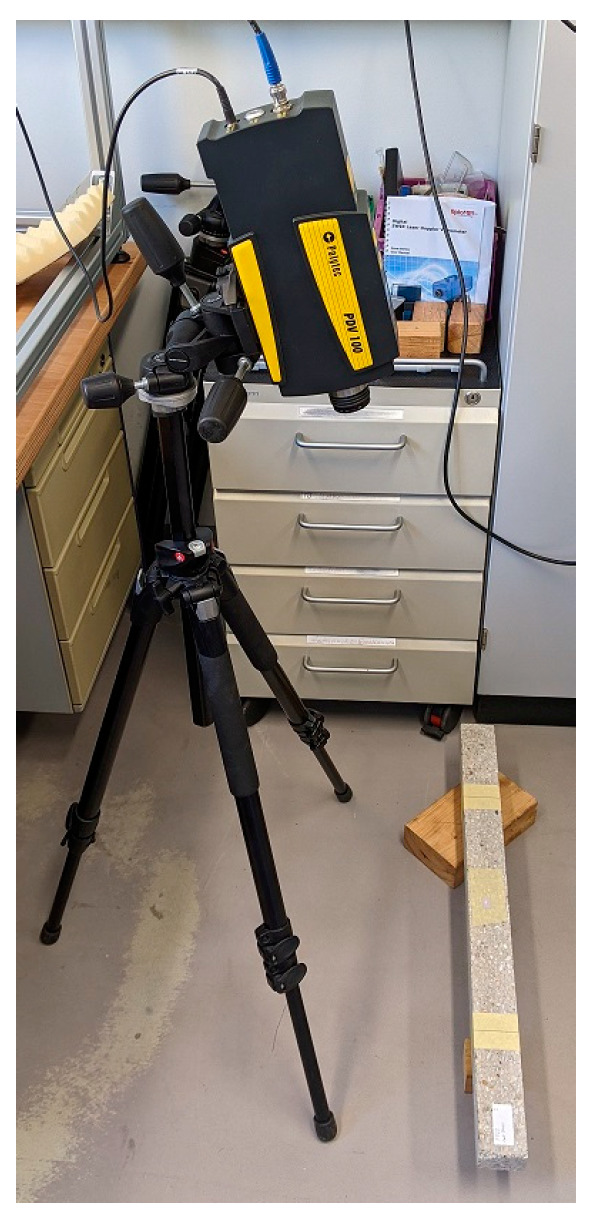
An overall view of the test setup.

The core-coating element was secured with adhesive tape at the mid-span (Figure 7). A single-point laser vibrometer was employed to measure the vibration (Brand: Polytec, Model: PDV-100). The monitoring point was at the mid-span of the beam. Figure 9 shows an overall view of the test setup.

### 3.2. Procedure of the Free Vibration Test

**Figure 10:**
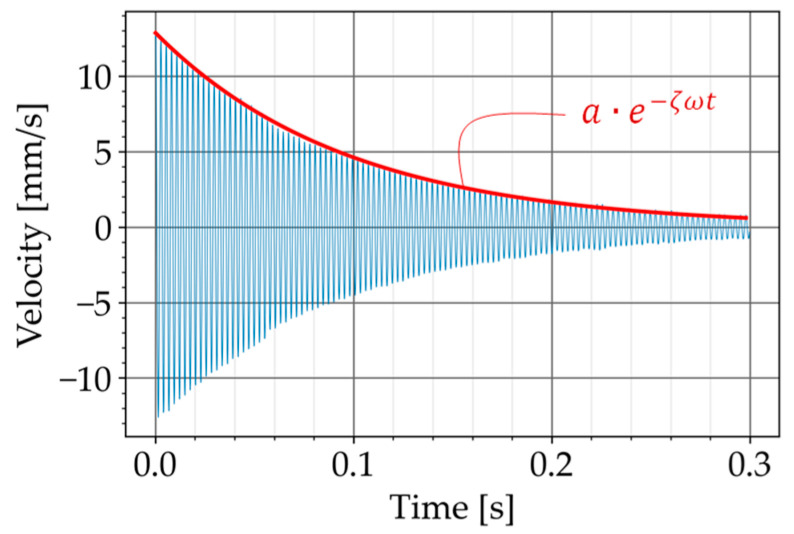
The decay of the free vibration and the fitting curve shown in red.

In the free vibration test, the beam was excited, approximately, at the mid-span with an impulse generated manually with an impact hammer (Brand: Sigmatest, Model: IH02). The velocity of the beam’s free vibration was then measured with the laser vibrometer. The monitoring point was at the beam’s mid-span. The output of this measurement was the decaying free vibration of the beam in the time domain (Figure 10).

To obtain the damping ratio, the exponential curve fitting method was used [21]. In this method, the decay of free vibration is approximated with the following exponential function

(2) $$a\;\cdot \;e^{-\zeta \omega t}$$

where a is the initial amplitude, ζ is the damping ratio, ω is the natural frequency of the specimen and t is the time.

**Figure 11:**
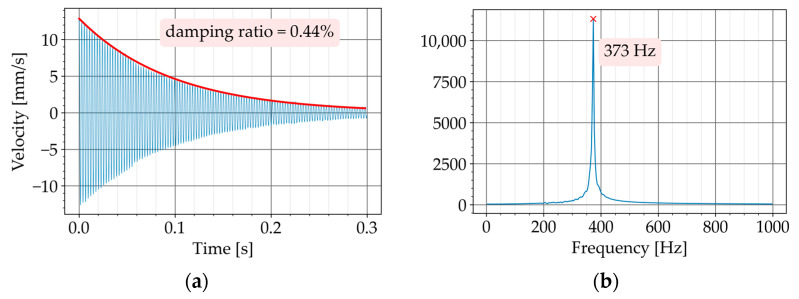

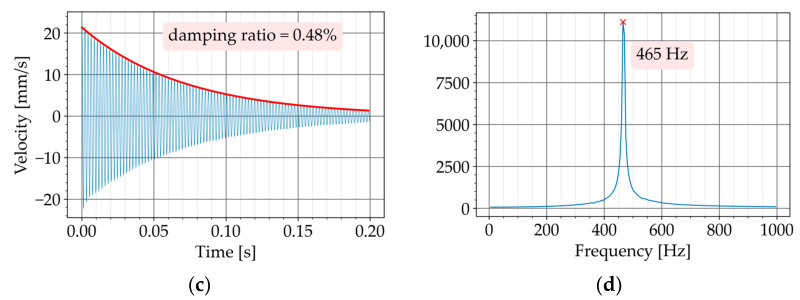
Decaying free vibration of the beams without the damper and their FFT diagrams (first step of the free vibration test). (**a**) P670: free vibration without damper. (**b**) P670: FFT of signal in a. (**c**) P600: free vibration without damper. (**d**) P600: FFT of signal in c.

The test had two steps. In the first step, the beam was tested without the core-coating inclusions. The natural frequency of the beam was determined with the Fourier transform of the free vibration in the time domain (Figure 11b,d). The natural frequency was necessary in Equation (2) for determining the damping ratio of the beam.

**Figure 12:**
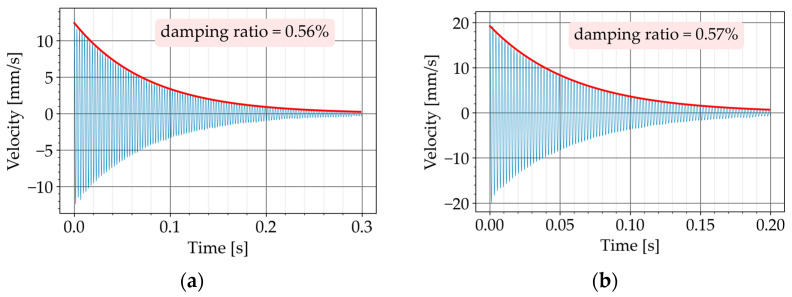
Decaying free vibration of the beams with the dampers (second step of the free vibration test). (**a**) P670: free vibration with damper 2 × K2. (**b**) P600: free vibration with damper 2 × K3.

In the second step, the inclusions were secured with adhesive tape at the mid-span of the beam (Figure 7 and Figure 8b). The test was repeated and the damping ratio was again determined (Figure 12a,b). By comparing the damping ratios, the impact of the inclusions was assessed.

### 3.3. Tuning the Inclusions

In the experiment, the core-coating inclusions were regarded as a classical TMD. Therefore, Den Hartog’s tuning parameters [1] were used for tuning the core-coating inclusions to the specimen. These parameters are two basic ratios.

Firstly, the mass ratio computed with

(3) $$\mu =\frac{m_{d}}{m_{m}}$$

where $m_{d}$ is the mass of the damper and $m_{m}$ is the mass of the main system. The effective mass of the main system in the mode under consideration should be used in the mass ratio [1]. Secondly, the frequency ratio computed with

(4) $$\kappa =\frac{f_{d}}{f_{m}}$$

where $f_{d}$ and $f_{m}$ are the natural frequencies of the damper and the main system respectively. When the mass ratio is known, the optimized frequency ratio can be calculated with Equation (5).

(5) $$\kappa_{opt}=\frac{1}{1+\mu }<1.0$$

The tuning parameters are presented in Table 4. The main mass for the tuning was the effective mass of the beam in the first mode. According to [1], the effective mass is a quarter of the total mass ($m_{m}$ in Table 4 is a quarter of m in Table 3).

Two core-coating configurations were used in the test as the TMD: K2 and K3 (Table 2). Their frequencies were determined in Section 2.2. The steel core was the oscillating mass of the damper. $m_{d}$ in Table 4 is the sum of the cores’ mass.

The current tuning parameters in Table 4 (μ and κ) were computed by using Equations (3) and (4). The optimized Kapa was calculated by substituting the current μ in Equation (5). Since the optimized tuning was not the goal of the experiment, the deviation from the optimized Kapa was disregarded. The damping ratio of the inclusions was neglected in this study too.

### 3.4. Results and Discussion

Table 5 summarizes the measured damping ratios. The test was repeated for every configuration a few times. The first column in Table 5 shows the test number. For every beam, the damping ratios are listed in two separate columns: for the beam alone (without damper), and for the beam with the core-coating configurations. The mean value of the measured damping ratios is provided in the last row.

The beam P670 exhibited an average damping ratio of 0.43% when it was tested alone. After securing two K2 configurations to P670, the damping ratio increased to an average value of 0.59%. This means an improvement of 37%.

The beam P600 exhibited an average damping ratio of 0.46% when it was tested alone. After securing two K3 configurations to P600, the damping ratio increased to an average value of 0.59%. This means an improvement of 28%.

For both beams, the damping ratios increased after securing the core-coating inclusions on the beam. A larger damping ratio signifies that the free vibration of the beam decays faster. This means that the core-coating inclusions could suppress the vibration of the beam by functioning as a damper.

## 4. Functioning as Multiple TMDs

The experiments in Section 3 demonstrated that the inclusions can function similarly to a classical TMD. In this study, the idea of the distributed core-coating inclusions in concrete is seen to be in line with the concept of a multi-TMD setup, which was reviewed in Section 1.2. This is because the inclusions are single TMDs distributed all over the vibrating body (Figure 2b). The authors of [6] provided that, in a classical multi-TMD setup, the natural frequencies of all TMDs are equal and tuned to a single natural frequency of the main system. Within the scope of this paper, the inclusions have identical diameters and configurations. This means that all of the inclusions theoretically exhibit an equal natural frequency and mass. In addition, they are tuned to a single mode of the main system. Therefore, they are regarded in this section as a classical multi-TMD setup.

The second objective of this study was to verify whether the randomly distributed inclusions could function similarly to a classical multi-TMD setup. To address this objective, numerical simulation and a Frequency Response Analysis (FRA) were used. The goal of the analysis was to obtain the response of a numerical model to harmonic excitation at a given frequency range. This method was also employed in other works, such as [5,6,7,8].

The expected result of the analysis was the transformation of the response curve from a one-peak to a two-peak response curve. The main system without the inclusions exhibit a response curve with one peak at the resonance vibration. If the inclusions function similarly to a multi-TMD setup, the peak at the resonance will be suppressed and two new peaks will be appeared before and after the resonance peak [5,6,7,8]. In this regard, two numerical models were required for every configuration in Table 6: one without the core-coating inclusions (normal concrete), and one with the inclusions (metaconcrete). The response curves of the models were then compared to verify the damping ability of the inclusions.

### 4.1. Description of the Numerical Model

The models were small-scaled beams with the given specifications in Table 6. The models were created at the mesoscale and composed of three phases: mortar matrix, particle phase, and the rigid Interfacial Transition Zone (ITZ) [22]. Table 7 summarizes the components of the meso-model and their material properties assumed in the simulation.

The mortar matrix is a mixture of cement, water, air, and aggregates with diameters smaller than 8 mm. Incorporating the aggregates smaller than 8 mm in the mortar matrix can minimize the required computational power [22]. The mortar matrix in the FE-Model was defined by assigning the material properties to the corresponding domain. These properties were mainly E and Density as provided in Table 7.

The particle phase contained the core-coating inclusions and the normal aggregate with diameters from 8 to 16 mm. The normal aggregates were assumed to have spherical shapes. The volume fraction of the core-coating inclusions in the model was chosen to be 5%. They replaced an equal fraction of the normal aggregate. Therefore, the total volume fraction of the particles in the models with and without the core-coating inclusions remained fixed at 28.4%.

An algorithm based on the Take-and-Place method [23] was programmed in Python to generate a random distribution of the normal aggregates and the core-coating inclusions. The algorithm was mainly based on the developed formulation in [22] with minor simplifications to suit this study. The code follows a rather simple procedure. Particles of different sizes were generated. They were placed one by one in a position that was randomly chosen from the volume of the specimen. By every placement, the particle was checked for overlap with the surrounding existing particles. If an overlap was detected, the particle was relocated. This procedure was repeated until the particle was placed without overlapping the surrounding particles [22,23].

To generate the meso-beam in Abaqus, a cuboid was first created, which represented the mortar matrix. The particles were then created inside the cuboids. The “Merge” function in the “Assembly” module of Abaqus was used to merge these two phases. Therefore, the phases were in direct contact without any voids between them. This resulted in a rigid ITZ between the mortar matrix and the particles. In the discretized FE-Model, the difference between the phases was only in the material properties assigned to them (Table 7).

**Figure 13:**
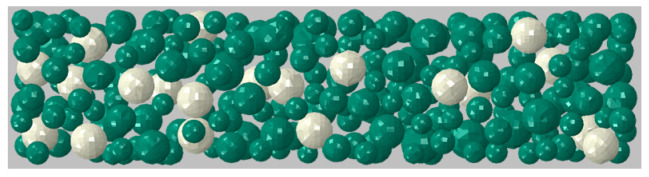
The side view of the internal structure of a meso-beam (transparent mortar matrix).

Figure 13 shows the internal view of the meso-beam. Only half of the beam was modeled in Abaqus. This compromised the random distribution of the particles in the second half, but significantly reduced the analysis time.

### 4.2. Boundary Conditions and Applied Load

**Figure 14:**
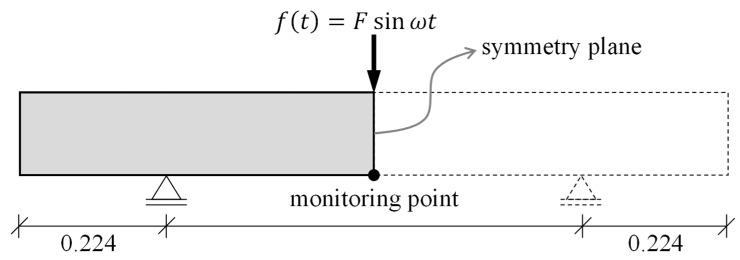
The boundary conditions of the numerical model and the applied load.

The beams were point-supported, similarly to the experiments in Section 3 (Figure 14). The distance between the supporting points in the 3D-Model was chosen based on the first vibratory mode shape of the beam with free ends. The Vertical displacement of the supporting point was restrained (U2 = 0). Since only half of the beam was modeled, the “symmetry boundary condition” in Abaqus was assigned to the plane at the mid-span of the beam (XSYMM: U1 = UR2 = UR3 = 0).

A harmonic point load with an amplitude of 1 N was applied at the mid-span of the beam. The frequency range of the load was within 500 to 1500 Hz. For the response curve of the model, the deformation at the mid-span was considered as the monitoring point (Figure 14). 

### 4.3. Analysis Procedure

The quadratic tetrahedral element of type C3D10 was used in the FE-Model. The global seed size was 5 mm and the “free” mesh technique in Abaqus was chosen to discretize the model.

The first step of the analysis was a “Natural frequency extraction” with “Lanczos” eigensolver in Abaqus. The target mode shape of the beam was found after the analysis by reviewing the extracted mode shapes. Table 6 provides the frequency of the target mode. This natural frequency was required for the tuning.

The second step of the analysis was a “direct steady-state dynamic analysis” in Abaqus. The lower and the upper frequencies of the harmonic point load were set to 500 and 1500 Hz, respectively. The number of points was chosen to be 200. This resulted in a frequency sweep of 5 Hz. The bias was set to 1. The analysis output was the response of the model to the harmonic excitation at the given frequency range. Ultimately, the response curves of the models with and without the inclusions were compared to verify the damping ability of the inclusions.

### 4.4. Tuning the Inclusions

Similar to Section 3, Den Hartog’s tuning parameters were used here. Two core-coating configurations were used in the simulation: K4 and K5 (Table 2). Their frequencies were determined in Section 2.2. The steel core was the oscillating mass of the dampers. $m_{d}$ in Table 8 is the sum of the cores’ mass. The inclusions were tuned to the first vibratory mode of the beam with the given frequency in Table 6. The natural frequencies of the beam were extracted in the first step of the analysis. As mentioned earlier, the volume fraction of the core-coating inclusions in the model was chosen to be 5%. By knowing the volume of the beam and the overall volume of one core-coating particle, the required number of them could be found.

The current tuning parameters are provided in Table 8. In contrast to Section 3, the total mass of the beam was considered here to compute the mass ratio. This is because the inclusions were evenly distributed in the entire beam. Similar to Section 3, the deviation from the optimized Kapa was disregarded.

### 4.5. Results and Discussion

**Figure 15:**
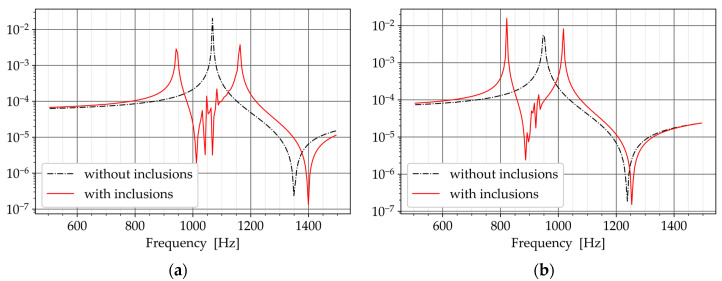
The response curve of the models with and without the inclusions. (**a**) P480. (**b**) P510.

Figure 15 compares the frequency response diagrams of the beams. The response curve of the beams without the inclusions exhibited one peak corresponding to the first vibratory mode (dash-dotted line in Figure 15a,b). In the response of the beams with the inclusions, the corresponding peak disappeared from the response curve and two new peaks appeared before and after the first mode (solid line in Figure 15a,b). This happened because the inclusions were tuned to the first vibratory mode of the beams. Therefore, they suppressed the response of the first mode.

The transformation of the response curve from a one-peak to a two-peak response verified that the inclusions could suppress the target mode by functioning as multiple TMDs. The transformation of the response curve was also used in [5,6,7,8] to evaluate the functionality of the multi-TMD setup.

The authors of [6] studied, in particular, the effect of the frequency range of multiple TMDs on the response curve. The two-peak response curve was observed when multiple TMDs had a frequency range narrower than 0.1 Hz. A narrow frequency range caused multiple TMDs to function like a single TMD. The core-coating inclusions with equal natural frequencies in this study resembled the narrow frequency range in [6]. Therefore, the two-peak response curve was the expected result that verifies the damping ability of the distributed inclusions.

## 5. Conclusions

Resonance vibration can increase the amplitude of the structure’s vibration obnoxiously. Using a damper tuned to the critical vibratory mode of the structure is a classical solution to avoid this unpleasant phenomenon. This study investigated the utilization of silicone-coated steel balls in concrete as damping aggregates to suppress the resonance vibration similar to a TMD. The engineered inclusions were randomly distributed in concrete and formed a multi-TMD setup.

The authors first proved the ability of the individual inclusions to function similar to a TMD through the free vibration test. The damping ratio of two small-scaled concrete beams was measured in the lab. The damping ratio climbed after securing individual core-coating elements to the beams. One of the beams exhibited a 37% and the other 28% increase in the damping ratio.

Secondly, the authors verified the ability of the randomly distributed inclusions in concrete to suppress the resonance vibration. For this purpose, the frequency response analysis in Abaqus was employed to obtain the response curve of the meso-model of two small-scaled beams. For every beam, two models were created: one representing conventional concrete and the other representing concrete with the inclusions (metaconcrete). The change in the peak of the response curve clearly showed that the core-coating inclusions could suppress the resonance vibration of the beams. This result implies that the randomly distributed inclusions in concrete can be used as damping aggregates to suppress the vibration of a concrete beam. However, to extend the result to the structural components in practice, the core-coating inclusions with lower frequencies are required. The frequencies of the core-coating inclusions used in this study were much higher than the structural components in practice. For the inclusions with lower frequencies, steel balls coated with softer silicone must be investigated in future work.

The core-coating inclusions in the numerical models in Section 4 were identical in size and frequency. Using the inclusions of different sizes and frequencies should be investigated in future work. Such a configuration can be beneficial in suppressing multiple vibrational modes of the main system.

The material damping of the coating (silicone) was neglected in this study. Determining the damping of silicone through laboratory experiments and considering it in the tuning of the inclusions can be investigated in future studies.

## Figures and Tables

**Table 1:** Material properties used in the analysis.

Component	Material	Density (kg/m^3^)	E (MPa)
Core	Stainless-Steel	8000	200,000
Coating	Silicone	1040	1
Cube	Concrete	2400	38,000

**Table 2:** The core-coating sizes used in this work.

Configuration	Steel Core		Silicone Coating	Overall		Analysis Result
	Diameter (mm)	m (g)	Thickness (mm)	Diameter (mm)	m (g)	f ^1^ (Hz)
K2	20	34	11	42	70	370
K3	20	34	7	34	51	460
K4	8	2.1	3	14	3.4	1015
K5	10	4.2	3	16	5.9	892

^1^ The translational natural frequency of the core.

**Table 3:** The specifications of the specimens used in the experiment.

Specimen	Length (mm)	Section (Width × Height)	m (g)	f ^1^ (Hz)
P670	670	60 × 40	3800	373
P600	600	60 × 40	3410	465

^1^ The first natural frequency of the beam.

**Table 4:** Tuning the core-coating elements.

Main Mass			Damper			Current Tuning Parameters		Equation (5)
Beam	$m_{m}$	$f_{m}$	Configuration	$m_{d}$ ^1^	$f_{d}$	μ	κ	$\kappa_{opt}$
P670	950	373	2 no. of K2	68	370	0.07	0.99	0.93
P600	853	465	2 no. of K3	68	460	0.08	0.99	0.93

^1^ The mass of the core multiplied with the no. of inclusions.

**Table 5:** Experiment results: damping ratio of the beams with/without the damper.

Test #	Damping Ratio—P670			Damping Ratio—P600		
	Without Damper	With 2 × K2	Change	Without Damper	With 2 × K3	Change
1	0.44%	0.56%		0.51%	0.59%	
2	0.39%	0.57%		0.48%	0.58%	
3	0.40%	0.65%		0.40%	0.57%	
4	0.40%	0.57%		-	0.62%	
5	0.46%	-		-	0.59%	
6	0.46%	-		-	-	
mean value	0.43%	0.59%	+37%	0.46%	0.59%	+28%

**Table 6:** The specifications of the models in the simulation.

Model	Length (mm)	Section (Width × Height)	m (g)	V (cm^3^)	f ^1^ (Hz)
P480	480	40 × 60	2640	1152	373
P510	510	40 × 60	2800	1224	465

^1^ The first natural frequency of the beam.

**Table 7:** The components of the meso-model as per the mix design of concrete.

Phase	Component	Volume Fraction (%)		E (GPa)	Density (kg/m^3^)
		Model without the Inclusions	Model with the Inclusions		
Mortar Matrix	Cement	11.6		38	2360
	Water	15.8			
	Air	2			
	Aggregate 0/2	23.4			
	Aggregate 2/8	19.2			
Particles	Aggregate 8/16	28.4	23.4	63.5	2600
	Core-Coating	0	5	-	-
	sum	100	100		

**Table 8:** Tuning the core-coating inclusions.

Main Mass			Damper			Current Tuning Parameters		Equation (5)
Beam	$m_{m}$	$f_{m}$	Configuration	$m_{d}$ ^1^	$f_{d}$	μ	κ	$\kappa_{opt}$
P480	2640	1070	40 no. of K4	84	1015	0.032	0.95	0.97
P510	2800	950	30 no. of K5	126	892	0.045	0.94	0.96

^1^ The mass of the core multiplied with the no. of inclusions.

## Data Availability

Not applicable.
